# 1,25-Dihydroxy Vitamin D_3_ Facilitates the M2 Polarization and *β*-Amyloid Uptake by Human Microglia in a TREM2-Dependent Manner

**DOI:** 10.1155/2023/3483411

**Published:** 2023-05-27

**Authors:** Vo Thuy Anh Thu, Thi Xoan Hoang, Jae Young Kim

**Affiliations:** Department of Life Science, Gachon University, Seongnam, Gyeonggi-do 13120, Republic of Korea

## Abstract

Alzheimer's disease (AD) is a neurodegenerative disorder characterized by dementia as the primary clinical symptom. The production and accumulation of aggregated *β*-amyloid (A*β*) in patient brain tissues is one of the hallmarks of AD pathogenesis. Microglia, brain-resident macrophages, produce inflammatory cytokines in response to A*β* oligomers or fibrils exacerbating A*β* pathology in AD. HMO6 cells were treated with A*β*42 in the presence or absence of 1,25-dihydroxy vitamin D_3_ (1,25(OH)_2_D_3_) to determine its potential immunomodulatory effects, and the expression of pro-/anti-inflammatory cytokines, M1/M2-associated markers, Toll-like receptors (TLRs), and triggering receptor expressed on myeloid cells 2 (TREM2) was examined. 1,25(OH)_2_D_3_ was found to suppress A*β*-induced expression of proinflammatory cytokines (TNF-*α*, IL-1*β*, and IL-6), M1 markers (CD86 and iNOS), and TLR2/4, whilst increasing the expression of anti-inflammatory cytokines (IL-4, IL-10, and CCL17) and M2 markers (CD206 and Arg-1). Furthermore, 1,25(OH)_2_D_3_ promoted TREM2 expression and A*β* uptake by HMO6 cells, and the enhancement of A*β* uptake and M2 polarization was revealed to be TREM2-dependent. The findings of this study suggest that 1,25(OH)_2_D_3_ facilitates M2 polarization and A*β* uptake in a TREM2-dependent manner.

## 1. Introduction

Alzheimer's disease (AD) is a neurodegenerative disorder that accounts for 60-80% of dementia cases worldwide and is becoming an increasingly severe socioeconomic problem [1]. The major causes of the AD are the extracellular accumulation of beta-amyloid (A*β*) plaques and the intracellular accumulation of neurofibrillary tangles containing hyperphosphorylated tau proteins [2]. The accumulation of A*β* peptide is associated with proteolytic cleavage of the membrane A*β* precursor protein (APP), which is cleaved by *β*- and *γ*-secretases to yield a 37-49 amino acid residue peptide, A*β* [3]. Among the several APP cleavage products that contribute to AD, the 40- and 42-amino acid forms (A*β*40 and A*β*42, respectively) are the main final A*β* products [4]. A*β*42 is the more toxic form, which is attributable to the additional two amino acids that lead to misfolding and subsequent self-aggregation [5]. The accumulation of the more insoluble A*β*42 aggregates functions as a trigger for AD pathogenesis [6]. Aggregated extracellular A*β* may stimulate the activation of microglia, which are brain-resident macrophages that play a pivotal role in AD-associated neuroinflammation [7]. Activated microglia produce proinflammatory cytokines that are typically detected surrounding extracellular A*β* plaques in the brains of AD patients [8], and overactivation of these microglia promotes the development of inflammatory injuries and exacerbates the AD-associated pathology [9]. Thus, microglia-mediated inflammation is considered a promising therapeutic target for the treatment of AD.

TREM2 (triggering receptor expressed on myeloid cells 2) is a cell surface receptor expressed by microglia that plays an important role in the central nervous system (CNS) inflammation [10], and mutations in the TREM2 gene have been linked to an increased risk of developing AD [11, 12]. Ectodomain of TREM2 binds to a variety of ligands, including bacterial polysaccharides, lipoproteins, phospholipids, and DNA [13]. When such ligands bind to TREM2, intracellular signaling pathways are activated, which contribute to modulating cellular functions such as increased cell survival [14, 15], phagocytosis [14], cell adhesion and migration, and cytokine secretion [14]. Microglial TREM2 also detects extracellular A*β*, and its structure and function have been identified to influence the clearance and aggregation of A*β* [16–18].

The active form of vitamin D, 1,25-dihydroxy vitamin D_3_ (1,25(OH)_2_D_3_), is essential for calcium absorption and bone mineralization and is involved in a variety of biological processes, including cell growth and differentiation and immune response [19]. 1,25(OH)_2_D_3_ regulates target gene expression by binding to a nuclear hormone receptor, the vitamin D receptor (VDR) [20]. Emerging evidence suggests that vitamin D is involved in the early development of animal brains [21] and that maternal vitamin D deficiency during pregnancy is correlated with cognitive development disorders of offspring [22]. Importantly, patients with neurodegenerative diseases, including AD, have lower vitamin D serum levels [23, 24], and recent clinical studies have indicated the potentially beneficial effects of vitamin D supplements in the prevention of cognitive decline in AD patients [25, 26].

In this study, we treated human microglial cells (HMO6) with A*β* to mimic in vivo microglial activation and neuroinflammation in response to A*β* accumulation, which are the main pathological features of AD, and examined the potential immunomodulatory effects of 1,25(OH)_2_D_3_ in vitro.

## 2. Materials and Methods

### 2.1. Chemicals and Antibodies

Synthetic A*β*_1−42_ peptides (DAEFRHDSGYEVHHQKLVFFAEDVGSNKGAIIGLMVGGVVIA) were purchased from LifeTein (Beijing, China); 1,25(OH)_2_D_3_ was purchased from Sigma-Aldrich (Sigma-Aldrich, Saint Louis, MO, USA); antibodies for TLR4, CD86, CD206, iNOS, and Arg-1 were obtained from Santa Cruz Biotechnology (Dallas, TX, USA); antibodies for TREM2 (B-3) and A*β* were purchased from BioLegend (San Diego, CA, USA); anti-TLR2 antibody was purchased from Novus Biologicals (Littleton, CO, USA); and TREM2 antibody (237920; R&D Systems, Minneapolis, MN, USA) was selected as blocking antibody according to a previous study [27].

### 2.2. Cell Culture and the Preparation of A*β* and 1,25(OH)_2_D_3_

An established human microglial cell line [28], HMO6 (accession number CVCL_5G94), was used for this study. Cells were maintained in Dulbecco's Modified Eagle's Medium (DMEM) supplemented with 10% heat-inactivated fetal bovine serum (FBS) and 1% antibiotic-antimycotic (Invitrogen Corp.), in a 5% CO_2_ at 37°C.

The A*β*_1−42_ peptides and 1,25(OH)_2_D_3_ were dissolved in 5 mM dimethyl sulfoxide (DMSO) to prepare stock solutions and stored at -20°C. Before use, aliquots of these stock solutions were diluted to the requisite concentrations using DMEM containing 1% antibiotic-antimycotic.

### 2.3. RNA Preparation and Real-Time Quantitative PCR

Total RNA was extracted using an easy-BLUE™ Total RNA Extraction Kit (iNtRON Biotechnology, Inc., Seongnam, Korea) according to the manufacturer's instructions. The concentration of the extracted RNA was measured using a MaestroNano MicroVolume Spectrophotometer (Maestrogen, Las Vegas, NV, USA). Aliquots (2 *μ*g) of the purified RNA were used for the synthesis of cDNA using a Hyperscript™ 2 × RT Master Mix (GeneAll Biotechnology, Seoul, Korea). Quantitative real-time PCR was performed on a Rotor-Gene system (Qiagen) using a QuantiSpeed SYBR NO-ROX kit (PhileKorea, Seoul, Korea). Amplifications were performed using the primer sets listed in Table 1. Sample normalization was performed using the human *GAPDH* gene as an endogenous control. For each sample, the relative abundance of the target mRNA was calculated from the -△cycle threshold (△C_t_) values of the target and endogenous *GAPDH* reference genes using the 2^−△△^ C_t_ method.

### 2.4. Flow Cytometry

To examine expression levels of the cell surface proteins TLR2, TLR4, CD86, CD206, and TREM2, cells were collected, washed with DPBS 2 times, incubated with primary antibody at 4°C for 30 min, and followed by phycoerythrin- (PE-) conjugated secondary antibodies at 4°C for 30 min. To determine intracellular levels of iNOS and Arg1, cells were treated with 4% formaldehyde and 1% Triton at room temperature for 20 min. Then, cells were stained with primary antibody at 4°C for 30 min, followed by staining with PE-conjugated secondary antibodies at 4°C for 30 min. Cells were resuspended in phosphate-buffered saline (PBS) and analyzed on a Cytomics FC500 MLP (Beckman Coulter Inc., Fullerton, CA, USA).

### 2.5. Enzyme-Linked Immunosorbent Assay (ELISA) for A*β* Measurements

The culture supernatant was collected and added to the 96-well culture plate overnight at 4°C. To prevent nonspecific binding of antigens and antibodies to the well, bovine serum albumin (BSA) 5% was used as a blocking agent. Quantification of soluble A*β*_1−42_ was performed using anti-A*β* primary antibody and followed by incubation with m-IgG*κ* BP-HRP secondary antibody at RT. Absorbance of ELISA test was detected at 450 nm using microplate reader.

### 2.6. Statistical Analysis

The experiments were conducted at least three times, and all data are independently showed as mean ± standard deviation (SD). Significant differences among groups were analyzed by one-way analysis of variance (ANOVA) followed by post hoc test using SPSS 12.0 for Windows. Differences were considered statistically significant at *p* value less than 0.05. *T*-test was applied to determine significance among the groups of A*β*42 uptake.

## 3. Results

### 3.1. 1,25(OH)_2_D_3_ Downregulates A*β*-Induced Proinflammatory Cytokines and Upregulates Anti-Inflammatory Cytokines

**Figure 1:**
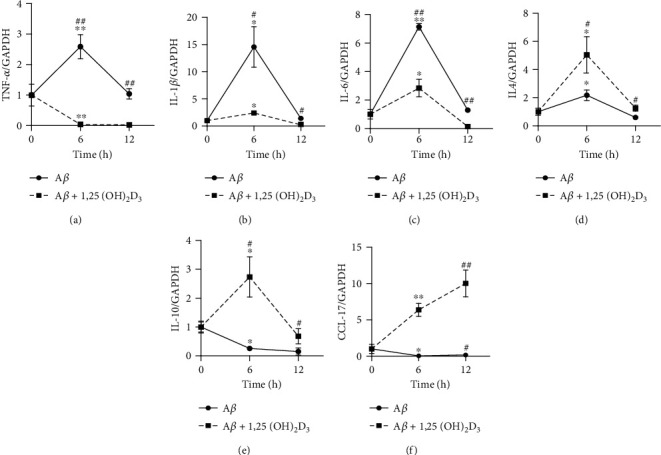
1,25(OH)_2_D_3_ downregulates A*β*-induced M1 cytokine expression, whilst upregulating M2 cytokine expression in HMO6 cells. Cells were incubated in DMEM exposed to 100 nM A*β*_1-42_ and treated with or without 1 *μ*M of 1,25(OH)_2_D_3_ for 6 h or 12 h. mRNA was extracted, and expression of proinflammatory cytokines TNF-*α* (a), IL-1*β* (b), and IL-6 (c) or anti-inflammatory cytokines IL-4 (d), IL-10 (e), and CCL17 (f) was measured by quantitative real-time PCR. ^∗^*p* < 0.05 and ^∗∗^*p* < 0.005 vs. 0 h control group; ^#^*p* < 0.05 and ^##^*p* < 0.005 vs. A*β*_1-42_-treated group.

Given that A*β* is an aggregation-prone peptide and that monomeric A*β* can be converted to not only stable oligomeric forms but also short fibrillar A*β* under physiologic solution conditions at higher A*β* concentrations [29, 30], we used monomeric A*β*42 to stimulate microglia in this study. Initially, we examined the effects of A*β*42 on the expression of selected cytokines in the HMO6 human microglial cell line. qRT-PCR analysis revealed that mRNA expression of the proinflammatory cytokines TNF-*α*, IL-1*β*, and IL-6 was significantly increased at 6 h after treatment with 100 nM A*β*42, but thereafter, expression declined to pretreatment levels at 12 h. However, the expression of these cytokines was markedly inhibited when the HMO6 cells were treated with both 100 nM A*β*42 and 1 *μ*M 1,25(OH)_2_D_3_ (Figures 1(a)–1(c)). Furthermore, we detected a significant reduction in the expression of the M2 cytokines IL-10 and CCL17 in A*β*42-treated cells, whereas that of IL-4 was increased. However, in response to the combined treatment with 100 nM A*β*42 and 1 *μ*M 1,25(OH)_2_D_3_, there were significant increases in the expression of these cytokines (Figures 1(d)–1(f)). These findings accordingly indicate that 1,25(OH)_2_D_3_ can reverse the proinflammatory cytokine expression induced by A*β*42 and enhance the expression of anti-inflammatory cytokines.

### 3.2. 1,25(OH)_2_D_3_ Reverses the A*β*-Induced Upregulation of TLR4 and TLR2 Expressions in HMO6 Cells

**Figure 2:**
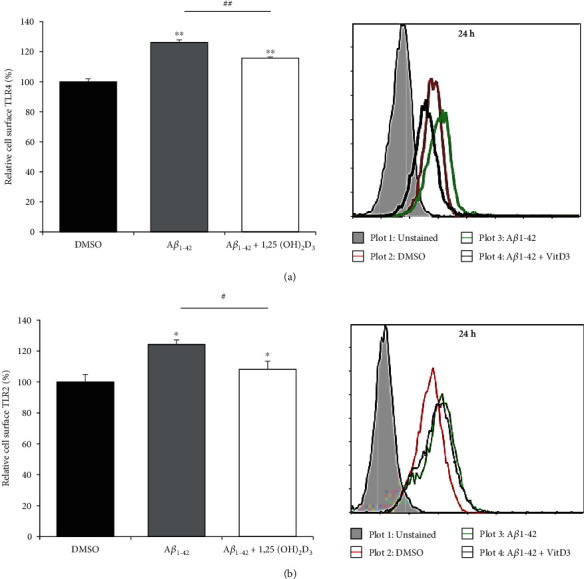
1,25(OH)_2_D_3_ downregulates the A*β*42-induced expression of TLR4 and TLR2 in HMO6 cells. Cells were treated with or without 1 *μ*M 1,25(OH)_2_D_3_ in the presence of 100 nM A*β*42 at 37°C for 24 h. Cell surface levels of TLR4 (a) and TLR2 (b) were measured by flow cytometry. Bar graphs indicate the relative expression ± SD (left panels). Right panels designate representative histograms. ^∗^*p* < 0.05 and ^∗∗^*p* < 0.0005 vs. the DMSO control. ^#^*p* < 0.05 and ^##^*p* < 0.0005.

Toll-like receptors (TLRs) are implicated in a range of neurological disorders, including AD, and are accordingly emerging as promising therapeutic targets for AD treatment [31]. Among the 10 human TLRs identified, TLR4 and TLR2 are reported to function as microglial A*β* receptors [32], which can induce proinflammatory cytokine production via the transcription factor activation of nuclear factor- (NF-) *κ*B in response to A*β* treatment [33]. We thus examined the surface expression of TLR4 and TLR2 on A*β*42-treated HMO6 cells. As shown in Figure 2, there was a significant enhancement of in the expression of these two receptors in A*β*42-treated cells, which was markedly reversed following treatment with 1 *μ*M 1,25(OH)_2_D_3_.

### 3.3. 1,25(OH)_2_D_3_ Downregulates A*β*-Induced M1 Marker Expression in HMO6 Cells, Whilst Upregulating M2 Marker Expression

**Figure 3:**
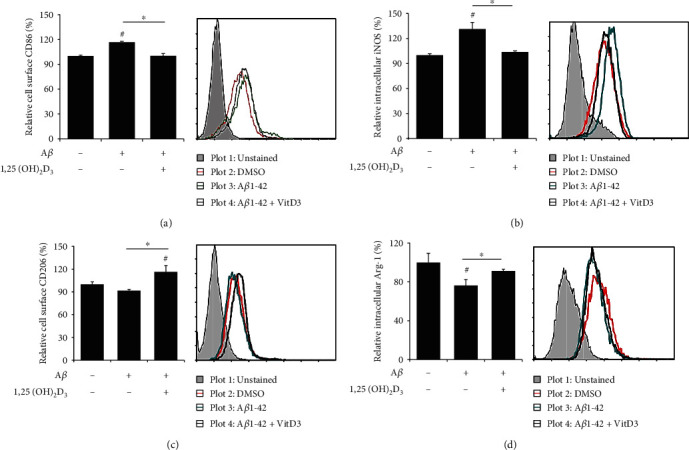
1,25(OH)_2_D_3_ downregulates A*β*42-induced M1 marker expression in HMO6 cells, whilst upregulating M2 marker expression. Cells were treated with or without 1 *μ*M 1,25(OH)_2_D_3_ in the presence of 100 nM A*β*42 for 12 h, and protein expressions of M1 markers CD86 (a) and iNOS (b) and M2 markers CD206 (c) and Arg-1 (d) were measured by flow cytometry. Bar graphs indicate the relative expression ± SD (left panels). Right panels designate representative histograms. ^∗^*p* < 0.05 vs. the DMSO control; ^#^*p* < 0.05 vs. the A*β*42-treated group.

Similar to macrophages, microglia can be classified into two distinct phenotypes based on their activation state, namely, M1 microglia, which produce proinflammatory cytokines, such as TNF-*α*, IL-1*ß*, and IL-6 [34], and contribute to inducing neuronal damage [35], and M2 microglia, which release anti-inflammatory cytokines, such as IL-4 and IL-10, and the Th2 chemokine CCL17 [36] and play role in neuroprotection [37]. In the present study, we examined the effects of A*β*42 treatment on the expression of M1 and M2 markers in HMO6 cells. In A*β*-treated cells, we detected a significant increase in the expression of the M1 markers CD86 and iNOS, which was reversed following treatment with 1,25(OH)_2_D_3_ (Figures 3(a) and 3(b)). Expression of the M2 marker arginase I (Arg-1), which metabolically dampens T-cell responses by causing arginine deprivation [38], in A*β*42-treated cells was significantly reduced, whereas that of CD206 remained unchanged. However, in cells cotreated with A*β*42 and 1,25(OH)_2_D_3_, we detected the markedly enhanced expression of both these M2 markers (Figures 3(c) and 3(d)). These findings thus provide evidence to indicate that A*β*42 treatment leads to M1 polarization in HMO6 cells and that 1,25(OH)_2_D_3_ skews the A*β*42-induced M1 phenotype toward an M2 phenotype in these cells.

### 3.4. 1,25(OH)_2_D_3_ Enhances Surface TREM2 Expression and A*β*42 Uptake by HMO6 Cells

**Figure 4:**
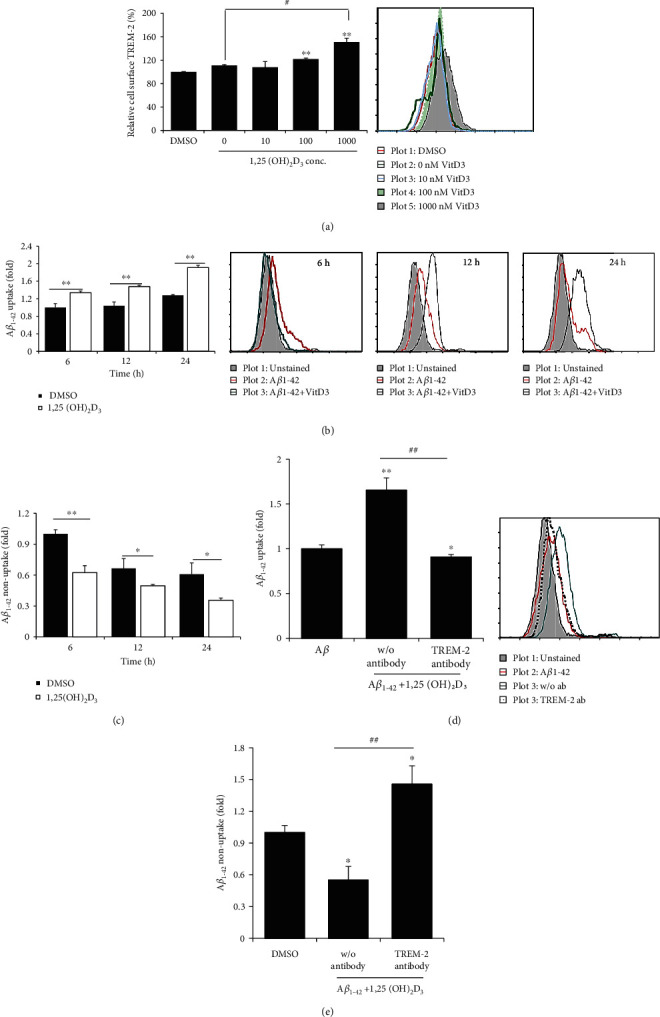
1,25(OH)_2_D_3_ enhances surface TREM2 expression and A*β*42 uptake by HMO6 cells. (a) Cells were treated with different concentrations of 1,25(OH)_2_D_3_ in the presence of 100 nM A*β*42 for 12 h, and cell surface expression of TREM2 was measured by flow cytometry. (b) Cells were treated with 100 nM A*β*42 in the presence or absence of 1 *μ*M 1,25(OH)_2_D_3_ at 37°C for different periods, and the levels of intracellular A*β* were measured by flow cytometry using anti-A*β* antibodies. (c) The culture supernatants were collected, and levels of A*β* were measured by ELISA. (d) Cells were treated with or without TREM2-blocking antibodies for 2 h and then treated with 100 nM A*β*42 in the presence or absence of 1 *μ*M 1,25(OH)_2_D_3_ at 37°C for 24 h, after which, the levels of intracellular A*β* were measured by flow cytometry. Bar graphs indicate the relative expression ± SD (left panels). Right panels designate representative histograms. (e) Culture supernatants were collected, and levels of A*β* were measured by ELISA. ^∗^*p* < 0.05 and ^∗∗^*p* < 0.005 vs. the DMSO control. ^##^*p* < 0.005.

As a cell surface receptor, TREM2 has been established to interact with A*β* and plays a central role in AD pathogenesis, and thus, we examined the effects of different concentrations of 1,25(OH)_2_D_3_ on the expression of TREM2 in HMO6 cells treated with 100 nM A*β*42. We accordingly found that when applied at concentrations of 100 and 1000 nM, 1,25(OH)_2_D_3_ promoted the enhanced expression of cell surface TREM2 in HMO6 cells treated with 100 nM A*β*42 (Figure 4(a)). To determine the capacity of human microglial cells to take up exogenous A*β*42 peptides, HMO6 cells were treated with 100 nM A*β*42 in the presence or absence of 1 *μ*M 1,25(OH)_2_D_3_ for different periods (6-24 h). Uptake of A*β*42 by HMO6 cells was determined by measuring the intracellular levels of A*β*42 by flow cytometry, and nonuptake of A*β*42 was accessed by measuring levels of A*β*42 in culture supernatants based on ELISA. The results revealed that at all assessed time points, the levels of A*β*42 uptake by HMO6 cells cultured in the presence of 1,25(OH)_2_D_3_ were higher than those in the absence of 1,25(OH)_2_D_3_ (Figure 4(b)). In line with expectation, nonuptake levels of A*β*42 in the culture supernatants of 1,25(OH)_2_D_3_-treated cells were significantly lower than those in the supernatants of nontreated cells (Figure 4(c)). These findings thus indicate that 1,25(OH)_2_D_3_ facilitates the uptake of exogenous A*β*42 by HMO6 cells. The phagocytosis of A*β* by microglial cells has been reported to be dependent on TREM2 [39], and thus, to determine the potential involvement of cell surface TREM2 in the 1,25(OH)_2_D_3_-enhanced uptake of exogenous A*β*42 by HMO6 cells, we used a TREM2-blocking antibody to block these receptors. In HMO6 cells pretreated with anti-TREM2 antibodies before treatment with A*β*42, we detected significantly lower levels of intracellular A*β*42 than in nontreated cells and even in DMSO-treated cells (Figure 4(d)). Correspondingly, thus nonuptake levels of A*β*42 in the culture supernatants of anti-TREM2 antibody-treated cells were significantly higher than those of nontreated cells and DMSO-treated cells (Figure 4(e)). These observations accordingly provide convincing evidence to indicate that cell surface TREM2 is involved in the 1,25(OH)_2_D_3_-enhanced uptake of exogenous A*β*42 by HMO6 cells.

### 3.5. 1,25(OH)_2_D_3_-Induced M2 Polarization Is Dependent on Cell Surface TREM2

**Figure 5:**
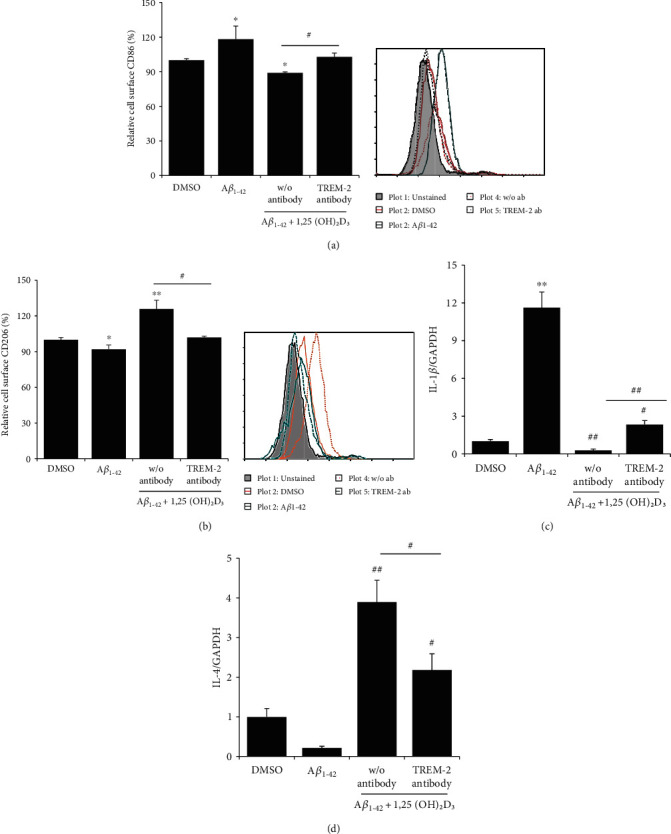
1,25(OH)_2_D_3_-induced M2 polarization of microglia is dependent on cell surface TREM2. HMO6 cells were pretreated with or without TREM2-blocking antibody for 2 h and then treated with or without 1 *μ*M 1,25(OH)_2_D_3_ in the presence of 100 nM A*β*42 at 37°C for 6 h (for mRNA expression) or 12 h (surface protein expression). Levels of cell surface CD86 (a) and CD206 (b) were measured by flow cytometry. Bar graphs indicate the relative expression ± SD (left panels). Right panels designate representative histograms. Levels of IL-1*β* (c) and IL-4 (d) mRNA expression were analyzed based on quantitative real-time PCR. ^∗^*p* < 0.05 and ^∗∗^*p* < 0.005 vs. the DMSO control; ^#^*p* < 0.05 and *^##^p* < 0.005 vs. the A*β*42-treated group.

Given that TREM2 has been proposed to play a vital role in the anti-inflammatory responses of microglia in AD [40], we examined the expression of M1/M2 markers and pro-/anti-inflammatory cytokines in HMO6 cells following stimulation with A*β*42 in the presence or absence of either 1,25(OH)_2_D_3_ or anti-TREM2-blocking antibody. Upon exposure to A*β*42, we detected a significant enhancement of M1 marker CD86 expression in HMO6 cells, whereas that of the M2 marker CD206 was reduced, and we found that both these responses could be reversed in the presence of 1,25(OH)_2_D_3_ (Figures 5(a) and 5(b)). However, the reduced expression of CD86 and enhanced expression of CD206 in 1,25(OH)_2_D_3_-treated cells were reversed in cells exposed to anti-TREM2-blocking antibodies. Similar to the expression patterns of M1/M2 markers detected upon challenge with A*β*42, the mRNA expression of IL-1*β* was significantly enhanced, whereas IL-4 was reduced in HMO6 cells, and these responses were reversed in the presence of 1,25(OH)_2_D_3_ (Figures 5(c) and 5(d)). Again, these 1,25(OH)_2_D_3_-mediated effects were reversed in the presence of anti-TREM2-blocking antibodies. These findings thus indicate that the cell surface receptor TREM2 may play a role in the anti-inflammatory responses of human microglial cells by facilitating M2 polarization.

## 4. Discussion

In this study, we demonstrated that in A*β*42-treated human microglial cells, the active form of vitamin D, 1,25(OH)_2_D_3_, promotes the downregulated expression of the proinflammatory cytokines TNF-*α*, IL-1*β*, and IL-6 and induces the upregulated expression of the anti-inflammatory cytokines IL-4, IL-10, and CCL17. In the CNS, microglia can recognize extracellular A*β* oligomers and fibrils and are thereby activated with the associated release of proinflammatory cytokines [41]. The sustained production of these cytokines from activated microglia can exacerbate the AD process [42]. In addition to their direct adverse effect on neighboring neurons [43, 44], the proinflammatory cytokines secreted by activated microglia can activate astrocytes that contribute to promoting neuronal loss in AD [45]. In this regard, the downregulation of proinflammatory cytokines and upregulation of anti-inflammatory cytokines induced by 1,25(OH)_2_D_3_ in A*β*42-treated HMO6 cells indicate that 1,25(OH)_2_D_3_ may have applicability in the treatment of AD. Interestingly, IL-4 was increased at 6 h after A*β* treatment in the absence of 1,25(OH)_2_D_3_. Mechanisms underlying the induction of IL-4 expression in microglia after A*β* treatment are currently unknown. Possible explanation for this induction is that IL-4 may be involved in counteracting the inflammatory response of microglia to A*β* or enhancing the phagocytic activity of microglia to promote the uptake and clearance of A*β* [46]. The application of 1,25(OH)_2_D_3_ has also been demonstrated to reduce the expression of TLR2 and TLR4, which are cell surface receptors for A*β*, the activation of which in turn induces microglial activation and neuroinflammation in AD [32]. The A*β*-induced activation of TLR2 and TLR4 promotes intracellular signaling leading to the production of proinflammatory cytokines. Consequently, the observed reduction in the expression of such proinflammatory cytokines in A*β*42-treated HMO6 cells in the presence of 1,25(OH)_2_D_3_ can probably be ascribed to the 1,25(OH)_2_D_3_-induced downregulation of TLR2 and TLR4 expressions.

Given that A*β* aggregates induce M1 microglial polarization [45] and that, in turn, the M1 inflammatory response led to the formation of A*β* aggregates [45, 47], it is important to facilitate either M2 polarization or A*β* clearance to suppress the progression of AD. In this regard, it is noteworthy that in A*β*42-treated HMO6 cells, 1,25(OH)_2_D_3_ downregulates M1 marker expression, whilst upregulating M2 marker expression, thereby indicating that 1,25(OH)_2_D_3_ has the effect of inducing M2 polarization. In this context, it has recently been suggested that promoting a phenotypic shift from M1 to M2 microglia may have therapeutic potential for the treatment of AD [48, 49], and accordingly, the 1,25(OH)_2_D_3_-induced polarization of a microglial phenotype from M1 to M2 could be considered as a promising therapeutic option.

Microglia play a central role in the clearance of A*β* aggregates via phagocytosis or receptor-mediated endocytosis, which are mediated by several types of cell surface receptors, including scavenger receptors, receptors for advanced glycation end products (RAGE), lipoprotein receptor-related proteins (LRPs), and TREM2 [50], among which TREM2 has been the most extensively studied. On the surface of microglial cells, TREM2 binds directly to A*β* oligomers with high affinity, and either the loss [51] or alteration [17] of TREM2 has been found to impair A*β* clearance by microglia. Consequently, it would appear that the maintenance of sufficient levels of TREM2 expression and function is important for retarding the progression of AD [52]. Our findings in this study revealed that 1,25(OH)_2_D_3_ enhances surface TREM2 expression and extracellular A*β*42 uptake by microglia and that the 1,25(OH)_2_D_3_-induced increase in microglial A*β*42 uptake is almost completely reversed in the presence of a TREM2-blocking antibody. These findings indicate that 1,25(OH)_2_D_3_ facilitates microglial A*β*42 uptake by upregulating the expression of cell surface TREM2. Consistent with this interpretation, the findings of a recent study have indicated that an agonistic TREM2 antibody, which can induce intracellular signaling, enhances the phagocytosis of oligomeric A*β* by microglia in vitro and improves cognitive function by attenuating chronic inflammatory responses in a murine model of AD [53, 54].

It is generally accepted that vitamin D plays an essential role in maintaining cognitive function in old age [55] and patients receiving hemodialysis [56], and the findings of several recent meta-analyses have indicated that lower serum levels of vitamin D are associated with a heightened risk of AD [57–60]. Moreover, human and animal studies have indicated that vitamin D contributes a reduction in A*β* deposition in the brain [61, 62]. Consequently, our observations in the present study, indicating that 1,25(OH)_2_D_3_ enhances the M2 polarization and A*β* uptake of human microglial cells in a TREM2-dependent manner, suggest a potential therapeutic option for the treatment of AD. However, more elaborate studies will be necessary to elucidate the precise mechanisms whereby vitamin D (1,25(OH)_2_D_3_)-enhanced TREM2 expression is involved in facilitating the M2 polarization and A*β* uptake of HMO6 cells. In addition, in vitro studies using primary microglial cells and animal AD model studies are needed to evaluate the potential clinical applications of 1,25(OH)_2_D_3_.

In conclusion, the findings of this study provide convincing evidence to indicate the potential protective effect of 1,25(OH)_2_D_3_ on A*β*42-treated human microglial cells by suppressing the expression levels of proinflammatory mediators, whilst enhancing the expression of anti-inflammatory mediators, which we suspect is associated with M2 polarization and A*β* uptake, plausibly mediated via an increase in cell surface TREM2 expression.

## Figures and Tables

**Table 1:** qRT-PCR primers used in this study.

Gene name	Forward primer (5′-3′)	Reverse primer (5′-3′)
GAPDH	ACAGCCTCAAGATCA TCAGCAAT	AGGAAATGAGCTTGACAAAGTGG
IL-1*β*	GGGATAACGAGGCTTA TGTGC	AGGTGGAGAGCTTTCAGTTCA
TNF-*α*	CAGAGGGCCTGTACCTCATC	GGAAGACCCCTCCCAGATAG
IL-6	GACCCAACCACAAATGCCAG	GAGTTGTCATGTCCTGCAGC
IL-4	CCGTAACAGACATCTTTGCTGCC	GAGTGTCCTTCTCATGGTGGCT
IL-10	TCTCCGAGATGCGTTCAGCAGA	TCAGA CAAGGCTTGGCAACCCA
CCL17	ACCCCAACAACAAGAGAGTGA	GAGGGCCCAGGTAGTCCC

## Data Availability

The data used to support the findings of this study are available from the corresponding author upon request.

## Abbreviations

1,25(OH)_2_D_3_: 1,25-Dihydroxy vitamin D_3_
AD: Alzheimer's disease
TREM2: Triggering receptor expressed on myeloid cells 2
TLR: Toll-like receptor
VDR: Vitamin D receptor.
